# Immunomodulation, Bioavailability and Safety of Bacteriocins

**DOI:** 10.3390/life13071521

**Published:** 2023-07-07

**Authors:** Svetlana V. Guryanova

**Affiliations:** 1 Shemyakin-Ovchinnikov Institute of Bioorganic Chemistry, Russian Academy of Sciences, 117997 Moscow, Russia; svgur@mail.ru; Tel.: +7-(915)-3150073; 2 Medical Institute, Peoples’ Friendship University of Russia (RUDN University) of the Ministry of Science and Higher Education of the Russian Federation, 117198 Moscow, Russia

**Keywords:** bacteriocins, immunomodulatory activity, innate immunity, inflammation regulation, microorganisms, lactic acid bacteria, nisin, sublancin, BacSp222, acidocin A, turicin H

## Abstract

The rise of antibiotic-resistant bacteria and the emergence of new pathogens have created a need for new strategies to fight against infectious diseases. One promising approach is the use of antimicrobial peptides produced by a certain species of bacteria, known as bacteriocins, which are active against other strains of the same or related species. Bacteriocins can help in the treatment and prevention of infectious diseases. Moreover, bacteriocins can be obtained in prokaryotic organisms, and contribute s to their widespread use. While the use of bacteriocins is currently limited to the food industry (for example, nisin is used as a preservative, E234), a large number of studies on their microbicidal properties suggest that their use in medicine may increase in the foreseeable future. However, for the successful use of bacteriocins in medicine, it is necessary to understand their effect on the immune system, especially in cases where immunity is weakened due to infectious processes, oncological, allergic, or autoimmune diseases. Studies on the immuno-modulatory activity of bacteriocins in animal models and human cells have revealed their ability to induce both pro-inflammatory and anti-inflammatory factors involved in the implementation of innate immunity. The influence of bacteriocins on acquired immunity is revealed by an increase in the number of T-lymphocytes with a simultaneous decrease in B-lymphocyte levels, which makes them attractive substances for reducing inflammation. The widespread use of bacteriocins in the food industry, their low toxicity, and their broad and narrow specificity are reasons for researchers to pay attention to their immunomodulatory properties and explore their medical applications. Inflammation regulation by bacteriocins can be used in the treatment of various pathologies. The aim of the review was to analyze scientific publications on the immunomodulatory activity, bioavailability, and safety of bacteriocins in order to use the data obtained to organize preclinical and clinical studies.

## 1. Introduction

Antibiotic resistance in bacteria is a global threat to healthcare systems. According to predictive statistical models, there were 4.95 million deaths associated with bacterial AMR in 2019, including 1.27 million deaths attributable to bacterial AMR [1]. Sub-Saharan Africa bore the highest burden of deaths, with 23.5 deaths per 100,000 population [2]. Deaths from hospital infections are increasing, with 42% of *Escherichia coli*, 35% of *Staphylococcus aureus*, and 8% of *Klebsiella* isolates in hospitals being resistant to antibiotics. Moreover, more than 20% of *E. coli* isolates, the most common causative agent of urinary tract infections, are resistant to both first-line drugs (ampicillin and co-trimoxazole) and second-line drugs (fluoroquinolones) [3,4]. Currently, over three million Americans suffer from infections caused by antibiotic-resistant strains [2]. If left unaddressed, deaths from antibiotic-resistant strains may become a leading cause by 2050, surpassing cancer and accounting for up to 10 million people per year [5].

The uncontrolled use of antibiotics in agriculture and medicine leads to the emergence of antibiotic-resistant bacterial pathogens in wastewater [6,7,8]. Residues of antibiotics used in agriculture exert selective pressure on bacterial communities in the soil–plant system, contributing to the emergence and spread of antibiotic resistance genes (ARGs) through horizontal gene transfer [9,10]. The spread of antibiotic resistance is also mediated by vesicles in the outer membrane of Gram-negative bacteria [11], conjugation between plasmids, transduction by bacteriophages, and natural transformation of extracellular DNA, which allows genetic material to move between strains and species [12].

To reduce the spread and emergence of new antibiotic-resistant strains, various approaches are being used to limit the use of antibiotics in agriculture [13,14] and medicine [15,16]. New control strategies are being developed, such as vaccines, antibodies [17,18], pattern recognition receptor ligands [19,20,21,22,23], probiotics, plant extracts [24,25], bacteriophages [26], cytokines [27], phytochemicals, silver and chitosan nanoparticles [28], antimicrobial enzymes, and peptides [29].

The use of bacteriocins, antimicrobial substances produced by bacteria, is one of the strategies to reduce extension of the antibiotic-resistant strains.

## 2. Resources, Gene Organization and Biosynthesis of Bacteriocins

Bacteriocins are peptide or protein antibiotics produced by bacteria that act only on strains of the same or closely related species [30]. Their history dates back to 1925, when André Gratia observed the inhibition of *Escherichia coli* ϕ by *E. coli* V [31], and Pierre Frédéricq [32] carefully described and introduced the concept of “colicin” for bacteriocins produced by *E. coli* [33]. Bacteriocins are now defined as peptides produced by bacteria, synthesized on ribosomes, which are active against other bacteria and against which the producer has a specific immunity mechanism [34]. Bacteriocins have several advantages, including low toxicity, a specific mechanism of action, and stability at low pH, high temperature, and over a wide range of salt concentrations [35,36]. They usually consist of 20–60 amino acids, with a positive charge and hydrophobic properties [37]. Another important trait of bacteriocins is their either linear or circular form. This has impacts on stability and activity. Bacteriocins can be degraded by proteases and digestive enzymes, limiting their oral use [38]. To preserve their activity, various delivery methods are being developed, such as liposomes [39], encapsulation [40], incorporation into coated tablets [41], starch-based matrices [42], and chemical modification of the C-terminus to protect against proteolytic enzymes [43,44].

Bacteriocins are produced by Gram-positive and Gram-negative bacteria, as well as archaea [45,46,47]. Some bacteria can produce several different bacteriocins [48,49,50,51,52] that are active against many pathogenic bacteria [53,54].

**Figure 1:**
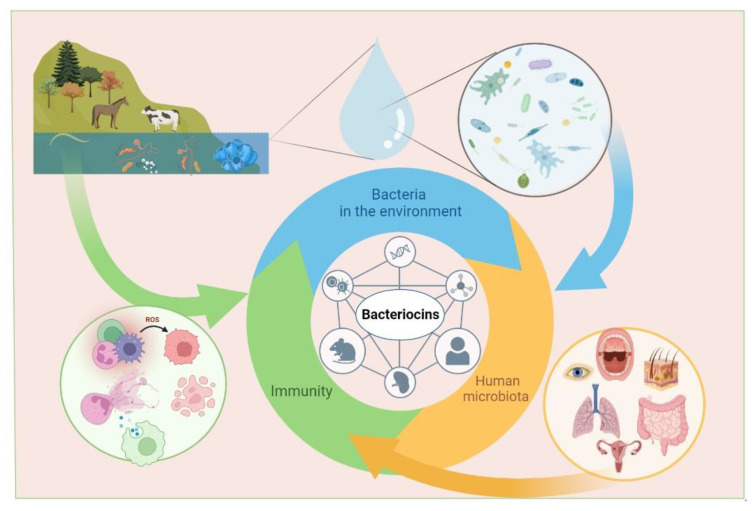
Bacteriocins are produced by bacteria living in water, soil, and in numerous organisms. Commensal microorganisms colonizing human skin and mucous and bacteria that enter human organisms from the environment also produce bacteriocins. Bacteriocins produced by human microbiota and by bacteria entering the human body from the environment affect human immunity by influencing on various populations of immune cells.

Bacteria that produce bacteriocins can be found everywhere: in water, soil, on the skin and mucous membranes of humans and animals, as well as in food and plants (Figure 1). The marine environment is considered to be one of the richest resources for the production of marine microbial bacteriocins [55]. Initially, researchers focused on studying sources such as food and animals [56,57]. In recent years, the bacteria Lactobacillus, Enterococcus, Streptococcus, and Staphylococcus, which inhabit the mucous membranes of the human oral cavity and gastrointestinal tract, have been extensively studied as potential sources of bioactive compounds, including bacteriocins [56,58].

Lactic acid bacteria (LAB) are known to produce various non-toxic bacteriocins that are pH stable and have a broad spectrum of antimicrobial activity. Bacteriocins of lactic acid bacteria do not adversely affect the intestinal microbiota as they are sensitive to the host’s digestive proteases, chymotrypsin, and trypsin [38,59]. The most common producers of bacteriocins in raw milk are Brevibacillus brevis and Bifidobacterium lactis; in yogurt and fermented soy products, *L. acidophilus* and *L. plantarum* are common, and in cheese and goat milk, *L. plantarum* is prevalent [60,61].

Several hundred bacteriocins have been described to date [62]. It is believed that 99% of all bacteria can produce bacteriocins, and many of them can produce more than one type, most of which are still unknown [59].

Bacteriocin genes can be located on both chromosomes and in a plasmid [63,64], with most colicin genes being found on plasmids [65]. These genes are typically arranged in clusters and contain one or more immunity proteins to prevent self-killing, and regulatory proteins provided post-translational modifications [66]. For instance, the CEA colicin gene is located on a plasmid; normally, its activity is suppressed by the LexA protein [67]. Subsequently, a high level of colicin causes the death of the producer cell. Interestingly, mutant bacteria without the LexA protein remain viable with an increase in colicin synthesis [67]. Cells that produce bacteriocins have multiple strategies to protect themselves from the lytic activity of their own bacteriocins. These strategies include the presence of specialized proteins or a system of pumps that remove bacteriocins from the cell, and sometimes a combination of these methods [68,69].

Bacteriocins are synthesized most often in the form of a precursor with further modification of the N-terminus [70]. However, circular, leaderless bacteriocins and colicins have no precursors [71,72]. Leaderless bacteriocins are active right after translation as they do not undergo any post-translational processing common to other groups of bacteriocins. Such leaderless bacteriocins may be attractive agents for medical use as their production becomes more accessible and cheaper [71].

Several databases have been developed to organize information regarding the structure, physicochemical properties, and mechanism of action of bacteriocins. One such database is BADASS, a user-friendly software package with a graphical interface that facilitates searching and analysis of bacteriocin diversity in full metagenomic shotgun sequencing data [73]. Another open-access database, BACTIBASE, provides detailed structural and functional information on 230 bacteriocins, including antimicrobial, physicochemical, and structural properties [74]. Other databases focus on specific pathologies and the regulation of immunocompetent cells by various substances, including bacteriocins, and visualize cause-and-effect relationships [75,76].

For instance, using a method of a random multiscale convolutional neural network, researchers found that potential bacteriocins could be HNH-endonuclease sequences from various marine bacteria. The Random Multiscale Convolutional Neural Network method, proposed by Cui Z. et al., establishes a random model for updating the scale value, and it offers a new classification system that predicts potential bacteriocin relationships [55]. Finally, state-of-the-art methods for predicting the efficacy of antimicrobial peptides, including bacteriocins, are being developed using deep learning (DL) approaches to optimize the search for new effective compounds [77].

## 3. Mechanism of Action on Microorganisms

Bacteriocins act in various ways, depending on their structural and physicochemical properties, as well as post-translational modifications. Most bacteriocins are cationic and interact with negatively charged bacterial membranes due to electrostatic forces, similarly to cationic antimicrobial peptides in multicellular organisms [78]. Electrostatic interactions occur between the negatively charged teichoic acids and phospholipids of Gram-positive bacteria, as well as with lipopolysaccharides of Gram-negative bacteria, and with Lipid II, which is a precursor molecule in the synthesis of the cell wall of both Gram-positive and Gram-negative bacteria [79]. This interaction represents the first stage in the manifestation of nonspecific and specific activity [34]. In the next stage, pores, ion-permeable channels, and cell death may occur [33], also due to the release of autolytic enzymes associated with lipoteichoic acids [80].

Another way for bacteriocins to enter the bacterial cell is by penetrating through a variety of transporters, which provide the necessary nutrients that ensure the vital activity of bacteria [81]. Some bacteriocins of Gram-positive bacteria, such as nisin, garvicin ML, lactococcin G, and sublancin 168, use zinc-dependent metallopeptidases, maltose ABC transporters, and undecaprenyl pyrophosphate phosphatase to enter the cell [81,82]. Gram-negative bacteria colicins use lipopolysaccharide (LPS) and transmembrane proteins in the outer membrane as receptors, allowing them to penetrate the cell through the two lipid membranes of the cell membrane. These transmembrane proteins are involved in the import of nutrients and metal ions, such as vitamins, sugars, and Fe^3+^. After penetrating the perioplasm, colicins interact with a translocator protein that helps establish an interaction with proteins called Tol or Ton in the host periplasm, followed by entry into the cell cytoplasm [83].

Interestingly, colicin-associated single-stranded DNA can also be transported into the bacterial periplasm. The discovered transport pathways of colicins can be utilized by bacteria to transport large molecules [84].

Studies have shown that bacteriocins from both Gram-positive and Gram-negative bacteria can use phosphotransferase sugar transport systems to enter the cell [34,85]. Once inside the bacterial cell, bacteriocins can cause the degradation of DNA and RNA, inhibit replication, transcription, and protein synthesis by specifically cleaving 16s rRNA, and stop the synthesis of the bacterial cell wall [86]. For instance, microcin C, microcin J25, and microcin B17 bind and inhibit Asp-tRNA synthetase, RNA polymerase, and DNA gyrase, respectively, which have been identified as their direct targets [45]. Ruminococcin C, isolated from the human microbiota bacterium Ruminococcus gnavus, interferes with the synthesis of nucleic acids without disrupting the membranes of target bacteria [87].

Despite the various possible mechanisms of antimicrobial strategies, the most common is pore formation in the target cell membrane [86]. Bacteriocins exhibit a bacteriostatic or bactericidal spectrum of action, mainly directed against bacteria closely related to the producing strain [88] and, in rare cases, against a wide range of bacteria [33]. It is worth noting that some bacteriocins use multiple types of mechanisms to influence the bacterial cell, significantly complicating bacterial resistance and the emergence of resistance.

Resistance to bacteriocins can be natural or acquired, appearing in previously susceptible strains [89,90]. Up to 8% of wild-type *Listeria monocytogenes* strains have been found to be naturally resistant to pediocin-like bacteriocins, particularly pediocin PA-1, while remaining sensitive to nisin [91,92]. Natural and acquired resistance to bacteriocins can emerge and disappear as a result of mutations in genes responsible for susceptibility [68]. For strains that do not produce bacteriocins, a common defense strategy is to synthesize extracellular proteases and peptidases [93]. For instance, *Enterococcus faecalis* secretes gelatinase to inactivate pediocin-like bacteriocins [94].

Acquired resistance can be developed through various strategies, such as increasing hydrophobicity to reduce membrane permeability, synthesizing the bacterial cell wall or cytoplasmic membrane [33,68]. For instance, *L. monocytogenes* can develop resistance to nisin by altering the composition of fatty acids that enter the membrane [95], while *Clostridium difficile* undergoes cell wall reorganization and changes in central cellular processes, such as carbohydrate metabolism, as well as an increase in the number of flagella per cell, without any change in cell mobility [96].

Moreover, certain bacterial strains can acquire multiple modes of resistance simultaneously, resulting in a shared resistance phenotype [97]. Understanding the mechanisms underlying the development of protection against bacteriocins is crucial for devising new strategies for the clinical use of bacteriocins, while also taking into account the potential adverse effects of resulting resistant strains.

## 4. Immunomodulatory Activity of Bacteriocins

Numerous studies have investigated the bactericidal, bacteriostatic, antiviral, antiparasitic, and antitumor properties of bacteriocins [33,38]. However, research on the immunomodulatory activity of bacteriocins is represented by few studies, making it difficult to characterize their overall immunomodulatory and allergenic effects. Nevertheless, these studies suggest that the biological activity of bacteriocins may be similar to that of human antimicrobial peptides, based on comparable physicochemical properties [98]. For instance, the lanthiobiotic nisin Z has been found to induce the secretion of chemokines MCP-1, Gro-α, and IL-6 in human peripheral blood mononuclear cells (PBMCs) in a dose-dependent manner (concentrations of nisin Z 50, 100, 150 µg/mL), wherein nisin Z significantly reduces TNF-α induction in response to bacterial LPS (concentration of 2 ng/mL) PBMCs. The inhibition of nisin Z-mediated IL-6 secretion was effected by the inhibition of mitogen-activated protein kinase (MAPK) signaling and specific receptor tyrosine kinases. The inhibition of p38 (SB-202190), MAPK kinase (MEK) (U-0126), components of the MAPK: extracellular signal-regulated kinases 1 and 2 (ERK1/2) pathway (c-Raf) ZM 336372, and the Src family kinases (PP1 and PP2) all significantly inhibited IL-6 induction by nisin Z. This suggested a strong correlation between ERK/MAPK signaling and the induction of immunomodulatory responses by nisin Z [99]. Furthermore, nisin Z has been shown to provide protection against both Gram-positive microorganisms *S. aureus* and Gram-negative *Salmonella enterica sv. Typhimurium* and *Escherichia coli* in mouse infection models. The authors of the study suggest that nisin Z modulates host immunity through mechanisms similar to those of human natural host defense peptides, involving multiple signal transduction pathways and growth factor receptors [99].

An increase in the levels of CD4 and CD8 T-lymphocytes was observed in mice with short-term dietary intake of nisin, along with a simultaneous decrease in B-lymphocyte levels. After long-term dieting, the number of T-cells returned to the control level. The period of administration of nisin was either 30, 75, or 100 days [100]. Nisin’s ability to lower the number of B-cells may prove useful in the treatment of periodontal and peri-implantitis [101,102,103], as increased amounts of T-cells are found in inflammatory infiltrate in peri-implant soft tissues [104]. Additionally, nisin was shown to disrupt oral pathogenic biofilms and restore microbiome composition towards healthy control levels in a peri-implantitis setting [101,102,103]. The authors conclude that nisin is a perspective agent in the treatment of periodontal and peri-implantitis inflammation [101,102,103].

Nisin is effective when used topically on mucous membranes due to its ability to restore the structure of the endometrium in a rat model and normalize the number of neutrophils to control levels, improve levels of IFN-γ, IL-2, IL-8, and inhibit the formation of *S. aureus* biofilms [103,104]. Additionally, nisin has been found to significantly reduce not only the levels of several periodontal pathogens but also bone loss and the oral and systemic inflammatory response of the host. At the same time, nisin increased the population of fibroblasts and osteoblasts and mediated the proliferation of human periodontal ligament cells in a dose-dependent manner by increasing the proliferation marker Ki-67 [105].

Nisin was found to significantly increase the survival of mesenchymal stem cells (MSCs) from human bone marrow in vitro [106]. MSCs are used for transplantation, but their low survival rate after transplantation is a significant disadvantage. The survival and anti-inflammatory effects of nisin were assessed by cultivating MSCs against the background of exposure to H_2_O_2_ or in a serum-free medium using MTT analysis, ELISA, and real-time PCR. It was discovered that 250 and 500 IU/mL of nisin had a significant anti-apoptotic effect on MSCs, increasing cell viability and proliferation. The expression of IL-10, fibroblast growth factor 2 (FGF-2) and transforming growth factor-β (TGF-β) genes, as well as the synthesis of TGF-β and FGF-2 proteins, increased, indicating that nisin can have anti-inflammatory effects. During wound healing therapy with mesenchymal stem cells (MSC), an important limitation is that the MSCs are sensitive and short-lived in stress conditions. Preconditioning is effective to increase cellular resistance and survival, and nisin is a good choice as nisin improves the stability of MSCs. Long-lived MSCs produce more anti-inflammatory and less inflammatory cytokines and growth factors, which help cell repair and differentiation into fibroblasts at the site of tissue damage [106]. Nisin showed opposite pro-inflammatory effects on unstimulated and stimulated porcine PBMCs [107]. Nisin in a concentration of 50 µg/mL exhibited the proliferative activity, increasing the production of IL-1β and IL-6 and increasing the percentage of CD4+ CD8+ T in porcine PBMCs. After cell stimulation with *E. coli*, nisin showed antiproliferative activity, decreased phagocytosis, and inhibited the synthesis of IL-6 [107].

The zoonotic pathogen *Staphylococcus pseudintermedius* 222, able to infect humans, produces the unmodified form of the bacteriocidin BacSp222, as well as two post-translationally modified forms via succinylation and the cleavage of formylmethionine [108]. The production of such modified forms occurs in response to environmental changes, protects the cells of the producing bacteria from auto-toxicity of the secreted bacteriocin and limits the pathogenicity of the strain [109]. A study investigated the effects of these three bacteriocins and nisin A, used as a reference, on murine monocyte-macrophage-like Il- and murine brain endothelial (MBE) cell lines, as well as human polymorphonuclear neutrophils (hPMN) [108]. The results demonstrated that all tested BacSp222 compounds increased NO production and the expression of inducible nitric oxide synthase (iNOS) in combination with IFN-gamma in monocyte/macrophage-like cell lines P388.D1 and RAW 264.7, and they do not potentiate NO production by endothelial cells. Furthermore, all natural forms of BacSp222, either alone or with IFN-gamma, stimulated the production of TNF-α, MCP-1, and IL-1α. When combined with IFN-γ, the levels of IL-10 and IL-27 were increased. This studies on murine monocyte-macrophage cells revealed that bacteriocin BacSp222 and its forms activated the NF-κB transcription factor, leading to the increased expression of proteins associated with inflammation, such as iNOS and TNFα. In contrast, the IFN-β production was increased after the exposure of the cells to all forms of bacteriocin in the presence of IFN-γ. However, nisin A did not cause any changes in the production of the cytokines studied (IL-1α, IL-1β, IL-6, IL-10, IL-12p70, IL-17A, IL-23, IL-27, GMCSF, IFN-β, TNF-α) and did not influence the NO production and iNOS expression regardless of the presence or absence of IFN-γ on murine monocyte-macrophage-like cell lines. In human neutrophils, all forms of BacSp222 bacteriocin upregulated IL-8, but they did not induce ROS production or the formation of neutrophilic extracellular networks. In contrast, nisin A did not stimulate IL-8 production by human PMNs. Nisin A (1 μM nisin A for 4 h) also did not induce ROS production or the formation of neutrophilic extracellular networks. BacSp222 enhanced the expression of the iNOS only in the macrophages but not in the endothelial cells. Notably, in all experiments, the deformylated bacteriocin exhibited lower activity compared to the other forms of the peptide. BacSp222 and its succinylated form could be recognized as a novel peptide inducer of NO production by immune cells [108]. Another bacteriocin, AS-48, produced by different strains of *Enterococcus*, decreased nitric oxide (NO) production induced by LPS (up to 13.5 µM) (>96.51 µg/mL) on RAW macrophages and demonstrated an absence of pro-inflammatory effects [110].

Interestingly, the effects of nisin A on the activation of neutrophils in the experiment of other researchers revealed the ability to form neutrophil extracellular traps (NETs) [111]. Nisin A’s ability to activate neutrophils (concentrations of 75 and 150 µM) was demonstrated using scanning electron microscopy and fluorescence microscopy to observe the formation of NETs, well known for their ability to neutralize virulence factors and destroy bacterial pathogens. In addition, the presence of nisin A increased the intracellular level of neutrophil superoxide, which is normally produced by activated NADPH oxidase and is a prerequisite for the formation of NETs (Table 1) [111].

Thus, a comparison of the data obtained by Śmiałek J. et al. and Begde D. et al. revealed that nisin A in concentrations of 1 µM did not have an influence on the NETs formation, while in concentrations of 75 µM and 150 µM, the formation of NETs was observed [108,111].

**Figure 2:**
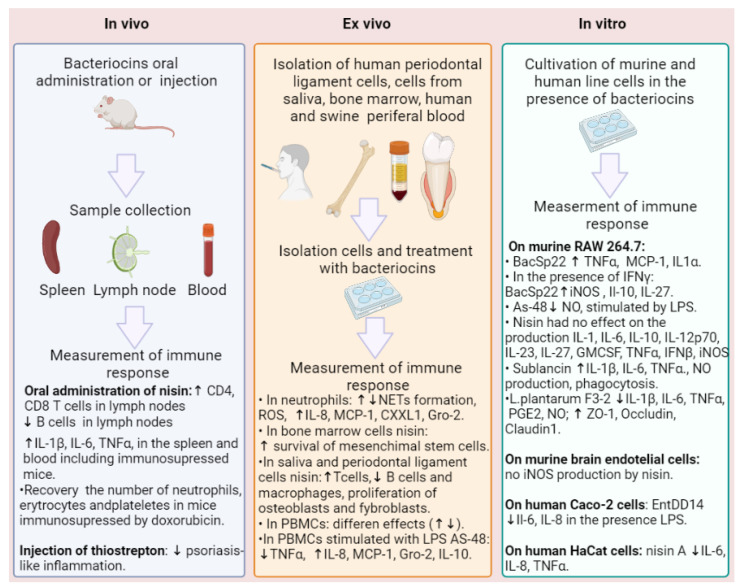
Investigation of the immunomodulatory activity of bacteriocins in vivo, ex vivo, and in vitro.

In a study examining the immunomodulatory activity of sublancin, isolated from *Bacillus subtilis* 168, an increase in CXCL1 and MCP-1 chemokine levels and a decrease in TNF-alpha production were observed in murine macrophage cells and neutrophils [112]. However, sublancin increased production of IL-1β, IL-6, TNF-alpha, nitric oxide, phagocytic, and microbicidal activity against MRSA in murine peritoneal macrophages and RAW264.7 cells (Figure 2) [113]. Oral administration of sublancin (1.0 mg/kg body weight) led to an increase in the expression of IL-1β, IL-6, and TNF-α mRNA in the spleen of BALB/c mice, including immunosuppressed mice treated with cyclophosphamide, and accelerated the recovery of peripheral leukocytes, erythrocytes, hemoglobin, and platelets while increasing the phagocytic activity of macrophages that had decreased after cyclophosphamide treatment. Together, these findings suggest that sublancin plays a crucial role in protecting against immunosuppression in mice treated with cyclophosphamide and may be a potential candidate for use in immunotherapy [114]. The study revealed that macrophage activation by sublancin is to some extent carried out through TLR4 with the participation of NF-κB and MAPK. When administered orally to mice, sublancin increased CD4 and CD8 T-lymphocytes in the mesenteric lymph nodes, indicating that it is capable of exerting an immunomodulatory effect by activating macrophage and T-cell immunity [113].

When studying bacteriocin-producing strains of *Lactiplantibacillus plantarum*, researchers found that *L. plantarum YRL45* significantly reduced the elevated levels of IL1β, IL6, TNFα, nitric oxide, and prostaglandin E2 induced by LPS in RAW264.7 cells, thereby reducing the severity of the inflammatory process [115]. Additionally, *L. plantarum* F3-2 significantly increased the expression levels of ZO-1, Occludin, and Claudin 1, all of which are involved in the formation of intercellular contacts in damaged cells of the intestinal epithelium, which is necessary to maintain the integrity of the epithelium [115].

On the other hand, acidocin A, produced by *Lactobacillus acidophilus TK9201*, was found to induce the production of a number of inflammatory mediators (IL-6, TNFα, MIG/CXCL9, MCP-1/CCL2, MCP-3/CCL7, and MIP-1β) in primary human monocytes, while also inhibiting the production of some anti-inflammatory factors such as IL-1RA and MDC/CCL22. This demonstrates its pronounced pro-inflammatory properties [116].

The enterocin DD14 (Ent DD14), which is produced by *Enterococcus faecalis* 14 strain isolated from newborn’s meconium, exerts the anti-inflammatory effect on the secretion of pro-inflammatory interleukins, including IL-6 and IL-8. The results show that EntDD14 is able to significantly decrease the secretion of both interleukins on Caco-2 cells following their treatments with LPS [117].

Thiostrepton is a ribosomally synthesized and post-translationally modified peptide (RiPP) produced by bacteria of *Streptomyces* genus [118,119]. Thiostrepton was reported to exhibit activity against Gram-positive bacteria and against various human cancer cells [120,121,122]. This bacteriocin has been characterized as a potent chemical inhibitor of the oncogenic transcription factor FoxM1, frequently overexpressed in cancers or other diseases [123,124,125]. The mechanism of action was defined through upregulation of heat shock proteins HspA1A, Hsp70, Hsp90α, or Hsp105 and triggering apoptosis in human cancer cells [126]. Thiostrepton inhibited TLR7-9 activation in mouse dendritic cells and did not inhibit NF-κB activation induced by TNF-α, IL-1, and other TLRs, as it inhibits TLR9 localization in endosomes via proteasome inhibition and via endosomal acidification. Moreover, in different murine models, thiostrepton attenuated LL37- and imiquimod-induced psoriasis-like inflammation. The researchers made the conclusion that thiostrepton is a novel TLR7-9 inhibitor, suggesting the potential therapeutic applications of thiostrepton on immunologic disorders elicited by inappropriate activation of TLR7-9 [127].

**Table 1:** Immunomodulation effects of bacteriocins.

Bacteriocin	Resource	Highlights	Ref.
Nisin Z	*L. lactis*	Inducing MCP-1, Gro-α, and IL-6 in human PBMC. In response to LPS, reducing TNF-α and inhibiting IL-6.	[99]
Nisin	*L. lactis*	Increasing T-cells, fibroblasts, and osteoblasts; increasing B cells. Increasing the expression of IL-10, FGF-2, and TGF-β genes and the synthesis of TGF-β and FGF-2. Decreasing phagocytosis and inhibiting the synthesis of IL-6.	[100,101,102,105,106,107]
Nisin A	*L. lactis*	No influence on IL-1α, IL-1β, IL-6, IL-10, IL-12p70, IL-17A, IL-23, IL-27, GMCSF, IFN-β, TNF-α, iNOS, NO production, and NETs.	[108]
		Inducing NETs formation and ROX in human neutrophils.	[111]
BacSp222	*Staphylococcus pseudintermedius* 222	Increasing NO, iNOS in P388.D1, and RAW 264.7; increasing IL-10 and IL-27 in combination with IFN-γ. In human neutrophils, upregulating IL-8; absence of ROS production or NETs formation.	[108]
AS-48	*Enterococcus* spp.	Decreasing NO production induced by LPS on RAW cells; absence of pro-inflammatory effects.	[110]
Sublancin	*Bacillus subtilis* 168	Increasing CXCL1 and MCP-1; decreasing TNF-α in murine peritoneal macrophages and neutrophils. Increasing IL-1β, IL-6, TNF-alpha, nitric oxide, and phagocytosis. Increasing T-cells in the mesenteric lymph nodes. Increasing IL-1β, IL-6, and TNF-α in the spleen of immunosuppressed BALB/c mice.	[112,113,114]
*L. plantarum* F3-2	*Lactiplantibacillus plantarum*	Reducing elevated levels of IL1β, IL6, TNFα, nitric oxide, and prostaglandin E2 induced by LPS in RAW264.7 cells. Increasing the expression levels of ZO-1, Occludin, and Claudin 1.	[115]
Acidocin A	*Lactobacillus acidophilus TK*9201	Increasing IL-6, TNFα, MIG/CXCL9, MCP-1/CCL2, MCP-3/CCL7, and MIP-1β in PBMCs.	[116]
Ent DD14	*Enterococcus faecalis* 14	Decreasing IL-6 and IL-8.	[117]
Thiostrepton	*Streptomyces genus*	Increasing HspA1A, Hsp70, Hsp90α, or Hsp105.	[127]

In addition to in vitro and in vivo studies, bacteriocins’ immunomodulatory activity is also being investigated through comparative genomic analysis. A study of genes from four *Lactobacillus* strains revealed that *L. plantarum SK151* had the highest number of genes with potential immunomodulatory activity—approximately 74. In *L. johnsonii PF01*, 41 genes were identified that covered both immune activation and immunosuppression, compared to *L. mucosae LM1* and *L. fermentum SK152*, which may be more effective in activating immune cells and the pro-inflammatory cascade than in suppressing it. Based on the similarities and differences between the four *Lactobacillus* species, the authors conclude that each strain’s immunomodulatory function should be experimentally studied and confirmed, since some genes’ activity may be strain specific and not identifiable through comparative genomics alone [128].

The immunomodulatory activity of bacteriocins is determined by their structural features, and the presence of N-formyl methionine contributes to the manifestation of pro-inflammatory activity. At the same time, the presence of N-formyl methionine is not essential for microbicide activity.

The effect of bacteriocins on immunocompetent cells is directly dependent on concentration. Using the example of bacteriocin nisin, a change in the activity profile from neutral to pro-inflammatory with increasing concentration was shown.

The variety of effects of bacteriocins on immune cells depends not only on the structure and concentration, but also on the context of their application. For instance, bacteriocin nisin in the absence of additional stimuli did not affect IL-8 and TNFα production, while in the presence of LPS, TNFα decreased and IL-8 increased. At the same time, the combined effect of nisin and IFNγ did not affect TNFα production.

Most bacteriocins, including nisin, lose their activity under the action of stomach and intestinal enzymes. However, it turned out that oral administration of bacteriocin nisin had an effect on the ratio of T- and B-cells in the spleen and lymph nodes of mice. The explanation could be that bacteriocin began to act already in the oral cavity, affecting immune organs.

**Figure 3:**
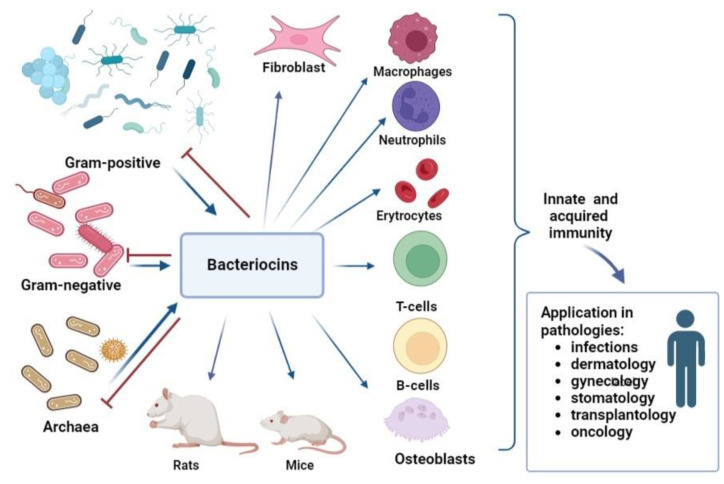
Bacteriocins resources, activities, and perspective of application. Bacteriocins derived from archaea, Gram-positive, and Gram-negative bacteria, may limit the viability of related strains by various means (indicated by red lines). In vivo studies in rodent models, as well as ex vivo and in vitro studies on various immune cells and cell lines, have shown the ability of bacteriocins to activate various subpopulations of immunocompetent cells, demonstrating an immunomodulatory ability (indicated by blue arrows).

Thus, the analysis of immunomodulatory ability showed that bacteriocins have an influence on innate immunity. For instance, some of bacteriocins can induce the production of reactive oxygen species, nitric oxide, phagocytosis, and NETs formation; others do not affect and even inhibit inflammation. The ability of bacteriocins to increase the number of T-lymphocytes, simultaneously decreasing B-lymphocytes and inducing cytokine and chemokine production, makes it possible to modulate acquired immunity too (Figure 3). Moreover, it is obvious that bacteriocins can regulate inflammation, inducing pro-inflammatory factors during infection or immunosuppression and suppressing pro-inflammatory cytokines in conditions of excessive inflammation.

An important factor that should be taken into account when using bacteriocins as antimicrobial agents is the release of the contents of bacterial cells during their destruction. A large number of biologically active compounds will affect immunocompetent cells, including through innate immunity receptors. The activation of innate immune receptors triggers intracellular pathways and alters immune response.

## 5. Bioavailability and Safety of Bacteriocins

Since the discovery of bacteriocins, researchers have mainly focused on determining their antimicrobial activity. However, bacteriocins can affect not only microbial communities but also eukaryotic cells. To use bacteriocins clinically as antimicrobial drugs, it is necessary to study their clinical efficacy, bioavailability and safety. To prevent side effects, it is also necessary to understand the mechanisms of action on tissues and organs when applied topically, as well as on immunocompetent cells when applied systemically.

To assess oral bioavailability, it is important to evaluate trans-epithelial transport and the effect of proteolytic enzymes in the gastrointestinal tract on maintaining the biological activity of bacteriocins. Nisin is a standard of comparison in the analysis of other bacteriocins due to its well-studied mechanism of action [129].

To analyze the possibility of migration of bacteriocins through epithelial cells of the gastrointestinal tract and vascular endothelial cells, the transport of fluorescently labeled bacteriocins—nisin, plantaricin 423, and bacST4SA—through colon adenocarcinoma cells (Caco-2) and human umbilical vein endothelial cells (HUVEC) was determined in vitro. It was found that after 3 h, 75% of nisin, 85% of plantaricin 423, and 82% of bacST4SA had migrated through the monolayer of Caco-2 cells. Over the same time period, 88% of nisin, 93% of plantaricin 423, and 91% of bacST4SA migrated through the HUVEC monolayer. It should be noted that the viability of both cell types remained unchanged when exposed to 50 μM of nisin, plantaricin 423, or bacST4SA. However, the effect of human plasma on the activity of bacteriocins depended on the structure and concentration of bacteriocins, and among the tested compounds, nisin was less stable. This study is one of the first to provide evidence that bacteriocins can cross the intestinal–hematological barrier [130].

When administered orally, it is crucial to evaluate the preservation of bacteriocin activity against gastrointestinal enzymes. Research conducted on laboratory and farm animals has shown that proteolytic enzymes in the stomach and small intestine, such as pepsin, trypsin, and chymotrypsin, cleave and inactivate many bacteriocins [131,132,133]. Bacteriocins without post-translational modifications are particularly sensitive to intestinal proteases, resulting in decreased antimicrobial activity when taken orally. An in vitro dynamic model examining the biostability of pediocin PA-1 under upper gastrointestinal conditions found that pediocin remained stable in the stomach but degraded completely when exposed to conditions equivalent to those found in the small intestine [134].

Bacteriocins with post-translational modifications are generally more resistant to proteases [135,136], but intestinal proteases can still inactivate and digest nisin A [137,138]. Initial research on the stability of microcin J25 by Pomares et al. (2009) showed that microcin J25 was resistant to digestion by proteolytic enzymes in the stomach and intestinal contents [139]. However, subsequent research conducted by Naimi et al. (2018) investigated the degradation of microcin J25 using both dynamic and static digestion models associated with antibacterial assays, LC-MS/MS, and molecular network analysis. The study found that while microcin J25 is remarkably stable under extreme conditions due to its lasso topology, it is degraded by the action of the pancreatic protease elastase and loses its antimicrobial activity [140]. To preserve the biological activity of bacteriocins, researchers are developing structural modifications using bioengineering methods, as well as delivery methods to the small and large intestines where bacteriocins act [41,42,44].

When bacteriocins are topically applied as part of hydrogels and nanoparticles, they can significantly increase the effectiveness of therapy in wound healing and combating infections of human and animal mucous membranes [104,141,142,143]. Bacteriocins, such as nisin, are also applied to titanium-based alloys, such as Ti6Al4V and its ultra-low interstitial version Ti6Al4V-ELI, which are commonly used for medical implants in orthopedics. Problems associated with infection, acute and chronic inflammation, osteolysis, and implant loosening and failure can be solved by applying bacteriocins to orthopedic materials [144]. Various solvents are being investigated to increase the solubility of bacteriocins. For example, an analysis of the effectiveness of nisin in promoting apoptosis of MG-63 osteosarcoma cells found that it was most effective in 0.05% acetic acid at concentrations of 800 μg/mL or higher, whereas in DMSO, and methanol at 0.05%, more than 90% of cells remained viable [145].

Safety studies have shown that bacteriocins have low toxicity and hemolytic activity. For example, the cytotoxicity of nisin is several times higher than the minimum inhibitory concentration [146,147]. In particular, an MTT-based cytotoxicity assay demonstrated nisin’s A cytotoxicity against human T-lymphoma Jurkat cells, Molt-4 cells, and freshly cultured human lymphocytes at concentrations greater than 200 µM (IC (50) 225 µM) [111].

Turicin H, which has inhibitory activity against *B. cereus*, *Bt Cry-B*, wild-type *E. faecium*, and *E. faecium ATCC*, but not *Bt Cry-B*/*pThurH* and *Bt Cry-B*/*pThurHΔThnA*, did not have hemolytic activity at a concentration of 20 µg/mL, which is four times higher than the minimum inhibitory concentration (MIC). This suggests the potentially safe use of turincin H as an antibacterial peptide for medical use [148]. While most bacteriocins are non-toxic to eukaryotic cells, enterococcal cytolysin has been found to be toxic [149]. Bacteriocin Ba49 from *Bacillus subtilis* subsp. *spizizenii strain Ba*49 present on the onion Allium cepa, showed low toxicity in three mammalian cell lines (HEK 293T, RAW 264.7, and L929) at concentrations several times higher than the MIC [150].

The bacteriocin AS-48, produced by *Enterococcus faecalis* and active against a number of Gram-positive bacteria, including *Mycobacterium tuberculosis*, did not show any cytotoxicity against macrophage cell lines THP-1, MHS, and J774.2 at concentrations close to its MIC [151]. The low cytotoxicity of AS-48, the absence of lymphocyte proliferation in vivo after skin sensitization in mice, and the lack of toxicity in a murine model support the consideration of the broad spectrum antimicrobial peptide AS-48 as a promising therapeutic agent for the control of a vast array of microbial infections, in particular, those involved in skin and soft tissue diseases [150]. Therefore, it is necessary to study the toxicity of bacteriocins to develop drugs based on them, as well as the methods and duration of application. It is also important to establish potential resistance to bacteriocins with repeated use [152].

## 6. Conclusions

Bacteriocins are not limited to acting on bacteria alone, as they exhibit activity against viruses, fungi, and parasites, and they also have an immunomodulatory effect on eukaryotic cells.

The limited number of scientific studies on the effects of bacteriocins on human immunocompetent cells restricts their potential use in medicine. However, the discovered properties of bacteriocins to modulate immunocompetent cells reveal their potential as immunomodulators. Furthermore, their cytotoxic activity against certain tumors presents opportunities for their use in complex therapy for oncology.

Multiple types of bacterial metabolites (quorum sensing molecules, pigments, antibiotics, etc.) have been proven to have immunomodulatory effects. The additional capabilities of bacteriocins to defeat pathogenic bacteria and concomitantly limit their inflammatory reactions provide support for applications of bacteriocins as therapeutic agents. Inflammation regulation by bacteriocins can be used in the treatment of various pathologies

Compared to other drugs, bacteriocins offer several advantages, such as high activity in the nano-molar range, low toxicity, and stability at low pH and high temperatures, as well as specific mechanisms of action. Additionally, probiotics can produce bacteriocins, which can be regulated in the gastrointestinal tract to prevent the spread of intestinal infections. This is particularly important in medical hospitals, where intestinal infections are a serious problem.

Research on both broad and narrow-spectrum bacteriocins can serve as a platform for developing complex and personalized therapies. Obtaining bacteriocins involves the use of prokaryotic systems and bioengineering methods, which significantly reduces production costs compared to eukaryotic antimicrobial peptides.

The widespread use of bacteriocins in the food industry, their low toxicity, and the presence of compounds with broad and narrow specificity make them a promising candidate for medical use. Therefore, researchers must pay attention to the immunomodulatory properties of bacteriocins to find opportunities for their medical use.

To determine the further directions of medical applications of bacteriocins, detailed studies on their effects on target cells and immunomodulatory activities on various populations of immunocompetent cells are necessary. Full-scale studies on the immunomodulatory activity of bacteriocins can expand the arsenal of drugs and optimize preventive and therapeutic strategies for precise medicine.

## Data Availability

Not applicable.
